# Prediction of small-molecule compound solubility in organic solvents by machine learning algorithms

**DOI:** 10.1186/s13321-021-00575-3

**Published:** 2021-12-11

**Authors:** Zhuyifan Ye, Defang Ouyang

**Affiliations:** State Key Laboratory of Quality Research in Chinese Medicine, Institute of Chinese Medical Sciences (ICMS), University of Macau, Macau, China

**Keywords:** Solubility prediction, Organic solvents, QSPR, Machine learning, lightGBM, Deep learning

## Abstract

Rapid solvent selection is of great significance in chemistry. However, solubility prediction remains a crucial challenge. This study aimed to develop machine learning models that can accurately predict compound solubility in organic solvents. A dataset containing 5081 experimental temperature and solubility data of compounds in organic solvents was extracted and standardized. Molecular fingerprints were selected to characterize structural features. lightGBM was compared with deep learning and traditional machine learning (PLS, Ridge regression, kNN, DT, ET, RF, SVM) to develop models for predicting solubility in organic solvents at different temperatures. Compared to other models, lightGBM exhibited significantly better overall generalization (logS  ± 0.20). For unseen solutes, our model gave a prediction accuracy (logS  ± 0.59) close to the expected noise level of experimental solubility data. lightGBM revealed the physicochemical relationship between solubility and structural features. Our method enables rapid solvent screening in chemistry and may be applied to solubility prediction in other solvents.

## Introduction

Organic solvents play an important role in the chemical industry. They are used in synthesis, catalysis, separation, quantitative analysis, and pharmaceutical formulation. Solvent selection depends on the solubility of compounds in multiple organic solvents. However, this has been a great challenge in chemistry and drug discovery. Since the last century, computational methods have been applied to estimating the solubility of compounds in water before the experiments [1–6]. These methods included mechanism models and QSPR (quantitative structure–property relationship) approaches. However, these methods are almost applied to prediction in water. There are relatively few approaches to predict the solubility in various organic solvents [7]. Currently, the solubility measurements still rely on laborious and costly experiments, which is an obstacle to rapid solvent screening for the chemical industry. Increasing attention has been paid to designing QSPR approaches to accurately predict the solubility of molecules in various organic solvents because it represents potentially massive time and materials saving.

Besides the QSPR approaches, some mechanism models have also been proposed for the solubility prediction in water or a single organic solvent since the last century. Table 1 summarizes the progress of mechanism models in solubility prediction. These methods include the simplistic rule-of-thumb “like dissolves like”, empirical equations, and thermodynamically-based mathematical expressions. Mathematical expressions were used to represent the correlations between solubility and other parameters quantitatively. For example, Hildebrand and Hansen solubility parameters are introduced to compute the solubility of drugs, excipients, and surfactants for dosage form design [8]. Flory–Huggins modeling calculates the entropy of mixing when mixing a polymer and a drug/solvent [9, 10]. The Flory–Huggins parameter χ determines the compounds miscibility and could be estimated by simulation methods or solubility parameters [11, 12]. Solvation Gibbs free energy is related to the solubility of substances. This is usually calculated by two types of thermodynamic cycles, involving the free energy of sublimation and solvating a gaseous molecule or the free energy of fusion and mixing. These cycles reflect the first-principles prediction and could be combined with machine learning models [13, 14]. If some physical constants, such as enthalpy of fusion and melting point, are available, it is also possible to predict ideal solubility. Then, the error between real and ideal solubility can be reduced by taking account of additional heat capacity, evidenced by the case of paracetamol [15]. As for the prediction in really nonideal solution, the activity coefficient can be modeled by the non-random two-liquid (NRTL) model, UNIQUAC, and UNIFAC methods [16–18]. The solubility in real solvents can be predicted by conductor-like screening model for real solvents (COSMO-RS), and it has been applied to drugs, pesticides, and asphaltenes [19–22]. Another mechanism model, molecular dynamics (MD), provides the dynamic evolution of a system, including structure, motion, and energy of molecules. It explains solubility through different aspects, such as Van der Waal forces and electrostatic forces between molecules [23]. Besides, fitted equations such as general solubility equations (GSE) are proposed by Yalkowsky et al. considering logP and melting point of a substance [24, 25]. logP is the measurement of the relationship between lipophilicity and hydrophilicity of a compound, which is determined by experiments.

**Table 1:** Progress of mechanism models in solubility prediction

Parameters	Methods	References
Solubility	Hildebrand and Hansen solubility parameters	[8]
Solubility	Fitted equations, such as GSE	[24, 25]
Solubility	COSMO-RS	[19–22]
Solubility	First-principles prediction	[13, 14]
Entropy of mixing	Flory–Huggins model	[9, 10]
Activity coefficient	NRTL, UNIQUAC, UNIFAC	[16–18]
Dynamic evolution	MD	[23]

A few studies predict the solubility in various organic solvents using these mechanism models. For example, in 2002, Gracin et al. predicted the solubilities of 9 compounds in water and 8 nonaqueous solvents using UNIFAC method [26]. In 2007, Eckert et al. applied COSMO-RS to estimating the solubility of 21 solutes in aqueous and nonaqueous solvents [27]. In 2011 and 2015, NRTL, UNIQUAC, UNIFAC, and COSMO-RS models were used to predict the solubilities of drugs and drug-like molecules in organic solvents by Matsuda et al. and Bouillot et al. [28, 29]. However, these conventional mechanism models required various experimental quantities for their implementation, such as enthalpy of fusion, Gibbs energy of fusion, and melting point. As a result, their practical application in solvents screening is still limited.

Machine learning algorithms have also been employed to construct models for solubility prediction in various organic solvents [7, 30, 31]. The models were generated on experimental solubility data. The accuracy of the models outperformed empirical equations and mechanistic models, such as UNIFAC and group contribution methods [7]. Linear equations and support vector machine (SVM) are two commonly used traditional linear and nonlinear methods. However, both methods do not suit well high-dimension nonlinear data featured in the task of solubility prediction. The dissolution process depends on many factors like solute lattice energy, solvation energy, solution interactions, and temperature. Additionally, traditional machine learning methods usually require reliable expertise to design features, but the expert’s knowledge and experience may be inadequate and subjective. On the other hand, complex molecular features and dissolution mechanisms also require automatic feature selection, which cannot be achieved in traditional methods. Besides, the previous studies only predict solubility in limited organic solvents, limiting their application in solvent screening. Therefore, there is still room for improvement of QSPR methods for predicting the solubility of compounds in various organic solvents.

Currently, advanced machine learning techniques as the core of data science and artificial intelligence have been successfully applied in many fields in academia, industry, and government [32–39]. Gradient boosting decision tree (GBDT) ensemble procedure framework has been widely employed [40]. light gradient boosting machine (lightGBM) based on GBDT can bundle and fuse mutually exclusive features to reduce the number of features [41]. lightGBM uses large separation points to build stable models for handling data with noise. In addition, recent development has been made through deep learning [often accepted as deep neural networks (DNN)]. The key aspect of deep learning is the general automatic feature extraction process. In the last 10 years, the application of lightGBM and deep learning in QSPR modeling can be found throughout aqueous solubility prediction and drug development with many successes. For example, in 2013, deep learning achieved good performance in aqueous solubility prediction based on molecular graphs compared to linear models, shallow neural networks, and SVM [5]. In 2019, the accuracy of the lightGBM model was found to surpass shallow neural networks, RF, and SVM in predicting the binding energy of cyclodextrins and guest molecules [42]. It is promising to use lightGBM and deep learning to accelerate solvent screening.

**Fig. 1:**
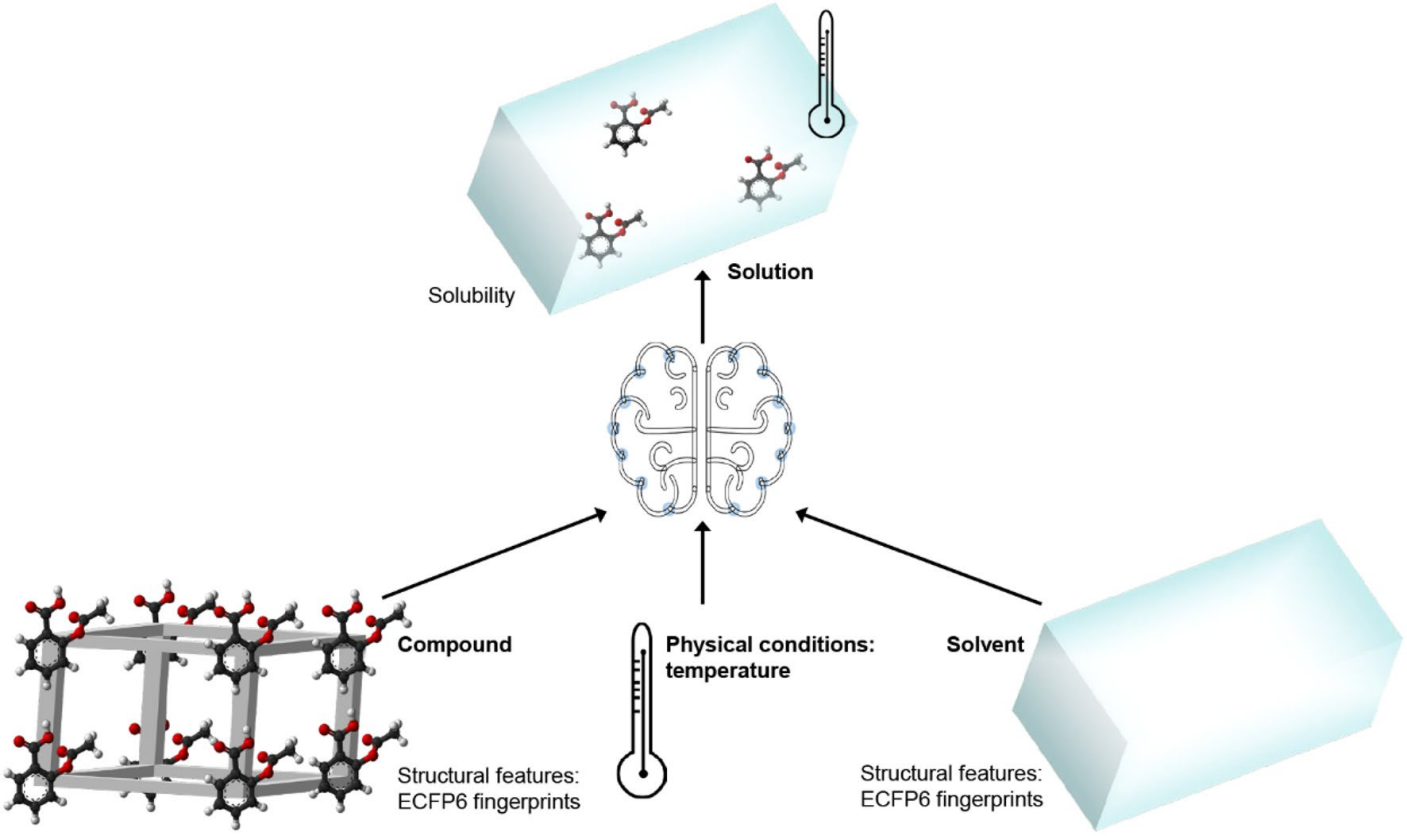
Concept of solubility prediction in organic solvents by machine learning

In this paper, machine learning models were developed to accurately predict the solubility of compounds in various organic solvents, which enabled rapid solvent screening. (Fig. 1) In detail, a dataset containing the experimental temperature and solubility data was collected to construct models. lightGBM, DNN, and other machine learning models were developed for good solubility prediction in organic solvents. For unseen solutes, the accuracy was analyzed. The relationship between structural features and organic solvent solubility was revealed.

## Methods

### Data collection

A dataset containing 5081 data on the solubility of compounds in various organic solvents at different temperatures was collected. 266 compounds and 123 organic solvents were covered in the solubility data. The data were extracted from literature. Each data entry records the solute, solvent, temperature at which the solubility was measured, experimental solubility, solubility unit, and data source.

The raw solubility data are presented in 12 solubility units, such as mole F, mol/F, and g/L. We collected the molecular weight and relative density of the compounds and solvents. Since the unit commonly used in published prediction studies is mol/L, the solubility unit of all data was standardized to mol/L. Ten data in volumetric fractions as solubility units were deleted. Later, considering the skewed distribution of the mol/L data, all solubilities were taken as logarithms to obtain normally distributed data and used as prediction labels for the models.

### Characterization of molecular structures

Extended-Connectivity Fingerprints (ECFPs) were applied to characterizing the structural information of compounds and solvents used in this study [43]. ECFPs and the temperature at which the solubilities were measured were used as inputs to models. ECFPs were designed for the characterization of structural features and the development of QSPR models. ECFPs have been widely used in the fields of cheminformatics and bioinformatics. In this study, ECFPs were generated by using the program RDKit [44]. The length and radius of ECFPs were set to 1024 and 3. The Canonical SMILES strings of compounds and organic solvents were retrieved from the PubChem database [45]. The SMILES were used to generate ECFPs. The ECFPs were calculated within seconds.

### Dataset splitting strategy

The complete dataset was split into two subsets of the training and validation subsets using random stratified sampling. The model was trained on the training subset of 4081 entries. The hyper-parameters of the model were adjusted on the validation subset of 1000 entries to find the best hyper-parameter configuration. Finally, the models were tested using a five-fold cross-validation method on the entire dataset. Based on the test result, the final generalization of the models was shown. This strategy for model development, validation, and testing was widely accepted in machine learning.

### Evaluation criteria

In machine learning, the coefficient of determination (R^2^) is a common metric used to evaluate the linear relationship between the true values and the predicted values of the model. In addition, mean absolute error (MAE) and mean squared error (MSE) were also commonly used as model performance metrics. MAE and MSE measured the mean of the absolute errors and the mean of the squared errors between the true values and the model predicted values on the data set. They are defined as follows:

$$MAE = \frac{{\mathop \sum \nolimits_{i = 1}^{n} \left| {\hat{y}_{i} - y_{i} } \right| }}{n}$$

$$MSE = \frac{{\mathop \sum \nolimits_{i = 1}^{n} \left( {\hat{y}_{i} - y_{i} } \right)^{2} }}{n}$$

$$R^{2} = 1 - \frac{{\mathop \sum \nolimits_{i = 1}^{n} \left( {\hat{y}_{i} - y_{i} } \right)^{2} }}{{\mathop \sum \nolimits_{i = 1}^{n} (y_{i} - \overline{y})^{2} }}$$

where n is the number of samples, $\hat{y}$ is the predicted value, *y*_*i*_ is the true value, $\overline{y}$ is the average value.

### Development of machine learning models

Recent advances in machine learning have been driven both by the availability of data in various domains and by developing learning algorithms and techniques. Various machine learning algorithms, such as linear regression, decision trees (DTs), SVM, neural networks, and others, have been proposed to estimate different types of mappings. This diversity of machine learning algorithms reflects the different application requirements. Different architectures and learning algorithms capture different types of mathematical structures and provide different trade-offs between computational complexity, data volume, and model performance. In addition, there are some generic modeling procedures, such as gradient boosting, that give final predictions by combining the outputs of multiple learning algorithms. In this study, DNN, SVM, DT, lightGBM, extra tree (ET), RF, partial least squared (PLS), k-nearest neighbors (kNN), and Ridge regression were used to predict the solubility of compounds in various organic solvents and at different temperatures. These learning algorithms incorporate linear and nonlinear methods, deep learning and shallow models, as well as ensemble learning methods and traditional learning methods such as linear regression and support vector machines. Specifically, DNN was built by using the Theano and Keras libraries [46, 47]. The lightGBM model was built using Microsoft's lightGBM package [41]. Other models were built by using the sci-kit learn package [48].

The hyper-parameter configurations of the learning algorithms have an important impact on the performance. The diversity of learning algorithms determines the variety of hyper-parameter search and tuning strategies. We trained the machine learning models on the training subset and adjusted the hyper-parameter configurations on the validation subset. Specifically, DT, kNN, and PLS used grid search because their hyper-parameters were discrete. lightGBM, ET, RF, and SVM used random search because their hyper-parameters were continuous and random search was a more efficient choice than grid search. Because of the large hyper-parameter search space of DNN, compared to other learning algorithms, the DNN models obtained using random search or grid search strategies may be under- or over-fitted. We manually adjusted the hyperparameters of the DNN by experts. The hyperparameter configurations of lightGBM (the learning rate, the number of trees, the subsample ratio, the subsample ratio of columns, the regularization terms of the number of leaves and the minimum number of samples in a child leaf), SVM (the kernel function, the penalty parameter C and the γ), RF (the number of the maximum features, the number of the trees, the regularization terms of the maximum depth, the minimum number of the samples used to split and the minimum number of samples in a child leaf), ET (the number of the maximum features, the number of the trees, the regularization terms of the maximum depth, the minimum number of the samples used to split and the minimum number of samples in a child leaf), kNN (the number of neighbors, the measurement of distance), DT (the regularization terms of the maximum depth, the minimum number of samples used to split and the minimum number of samples in a child leaf), PLS (the number of components), DNN (the number of hidden layers, the number of neurons in each of the hidden layers, the learning rate, the optimization algorithm, the number of epochs, the batch size, and the coefficient lambda of the l2 regularization) were listed in Table 2. The alpha of Ridge regression was set to 1.

**Table 2:** Model hyperparameter configurations of machine learning models

Machine learning algorithms	Hyperparameter configurations
lightGBM	0.1503; 486; 0.5338; 0.2431; 32; 18
SVM	Radial basis function; 112.4; 0.0006046
RF	486; 203; 45; 4; 2
ET	396, 504, 39, 4, 2
kNN	4; the standard Euclidean distance
DT	25; 8; 3
PLS	22
DNN	10; 1024, 256, 128, 64, 64, 64, 64, 64, 64, 64; 0.001; Adam with β1, β2 of 0.9, 0.999; 100; 500; 0.0003
Ridge regression	1

“;” Separates different hyperparameters

“,” The hyperparameter is composed of more than one element

## Results

### Data distribution

**Fig. 2:**
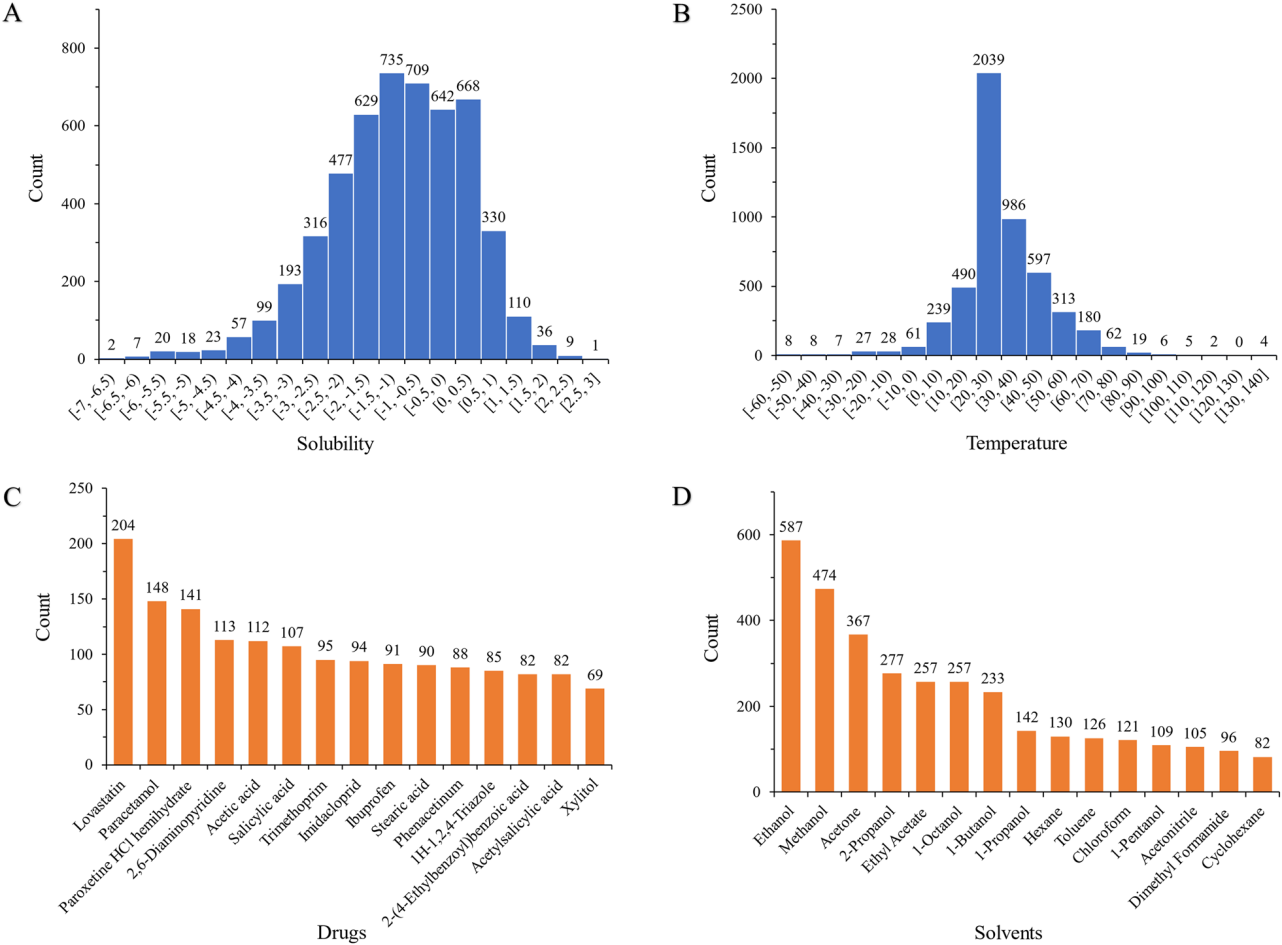
Solubility data (**A**), temperature (**B**), drug (**C**) and organic solvent (**D**) distribution in the solubility dataset

Solubility is used to reflect the maximum concentration of a solute dissolved in a solvent. In this study, 5081 solubility data were shown in the histogram chart range from − 7 to 3 in the solubility dataset (Fig. 2A). In the dataset, the solubility data in the logarithm of mol/L were not evenly distributed. The numerical value of the solubility varies widely. In general, compounds with solubilities less than or equal to 0 logarithm of mol/L are considered poorly soluble. According to the statistical results, the solubility data distribution in the dataset (Fig. 2A) was mainly located in − 3 to 1 logarithm of mol/L solubility range with 88.68% of total data volume in the dataset. This is possible because of the low solubility of the compounds discovered by modern chemistry methods of high-throughput screening and combinatorial chemistry. In addition, there was only a small fraction of solubility data located in − 7to − 5 logarithm of mol/L solubility range in the dataset, probably because compounds with solubility smaller than − 5 logarithm of mol/L was difficult to determine. These very poor soluble compounds tend to precipitate out of solution as small crystals in the systems. The solubility data distribution also demonstrates that the selection of organic solvents is an issue always concerned by chemical researchers since they displayed great challenges of organic solvent selection for the synthesized, separation, and formulation of compounds.

The experimental temperature data given in Celsius degrees were shown in the histogram chart (Fig. 2B). The experimental temperature data were not evenly distributed. Based on the statistical results, the experimental temperature data distribution in the dataset (Fig. 2B) was mainly located in the 0–60 °C range with 91.79% of total data volume in the dataset. There was obviously a high density of temperature data located in the 20–30 °C range in the dataset because the solubility of compounds in organic solvents was commonly determined at room temperature. There is no doubt that accurate prediction of solubility at room temperature is very important since most solubilities are measured in this experimental temperature range. Moreover, when the temperature is not in the range of 20–30 °C, it is challenging to build models with good performance in adapting to temperature changes.

The statistical results showed that the top 15 compounds covered 31.51% of solubility data in the solubility dataset (Fig. 2C). 66.19% of the data belonged to the top 15 organic solvents (Fig. 2D). A majority of compounds (142 in 266) in this dataset contained less than 10 solubility data. Similarly, many solvents (60 in 123) in this data set included less than 10 solubility data. This result demonstrated that the solubility data of compounds dissolved in organic solvents had a long tail. Predicting small sample data will be a challenge.

### Performances of different machine learning algorithms

In this study, nine machine learning algorithms (PLS, Ridge regression, kNN, DT, SVM, ET, RF, lightGBM, and DNN) were introduced to develop the models. Table 3 showed the accuracies of the nine machine learning models on the training and the validation subsets and in the final five-fold cross-validation test. In the final test, for R^2^, the accuracies of the nine machine learning models were roughly 0.8, with the lowest accuracy of 0.7382 ± 0.0122 for PLS, and the highest accuracy of 0.9192 ± 0.0115 for DNN. For MSE, the accuracies of the nine machine learning models were roughly 0.3, with the lowest accuracy of 0.4636 ± 0.0171 for PLS, and the highest accuracy of 0.1522 ± 0.0110 for lightGBM. For MAE, the accuracies of the nine machine learning models were roughly 0.3, with the lowest accuracy of 0.4908 ± 0.0125 for PLS, and the highest accuracy of 0.2029 ± 0.0074 for lightGBM.

**Table 3:** Performance of machine learning algorithms for prediction of the solubility of compounds in organic solvents

Machine learning algorithms	MAE			MSE			R^2^		
	Hold out		Five-fold cross-validation	Hold out		Five-fold cross-validation	Hold out		Five-fold cross-validation
	Training set	Validation set	Mean ± Std.	Training set	Validation set	Mean ± Std.	Training set	Validation set	Mean ± Std.
PLS	0.4508	0.4808	0.4908 ± 0.0125	0.3889	0.4541	0.4636 ± 0.0171	0.7806	0.7444	0.7382 ± 0.0122
Ridge regression	0.4459	0.4748	0.4845 ± 0.0122	0.3843	0.4470	0.4511 ± 0.0165	0.7832	0.7484	0.7454 ± 0.0095
kNN	0.2185	0.3382	0.3300 ± 0.0080	0.2089	0.4260	0.4142 ± 0.0232	0.8822	0.7602	0.7662 ± 0.0145
DT	0.2098	0.3343	0.3391 ± 0.0117	0.1368	0.3153	0.3443 ± 0.0310	0.9229	0.8225	0.8059 ± 0.0147
ET	0.1916	0.2745	0.2757 ± 0.0066	0.1085	0.2210	0.2286 ± 0.0134	0.9388	0.8756	0.8710 ± 0.0063
RF	0.1541	0.2443	0.2466 ± 0.0083	0.0900	0.2008	0.2147 ± 0.0126	0.9492	0.8870	0.8789 ± 0.0060
SVM	0.0954	0.2168	0.2173 ± 0.0112	0.0364	0.2086	0.1996 ± 0.0190	0.9795	0.8826	0.8873 ± 0.0106
DNN	0.0960	0.2102	0.2124 ± 0.0294	0.0335	0.1766	0.1548 ± 0.0215	0.9811	0.9006	0.9192 ± 0.0115
lightGBM	0.1085	0.2027	0.2029 ± 0.0074	0.0389	0.1554	0.1522 ± 0.0110	0.9781	0.9125	0.9142 ± 0.0053

**Fig. 3:**
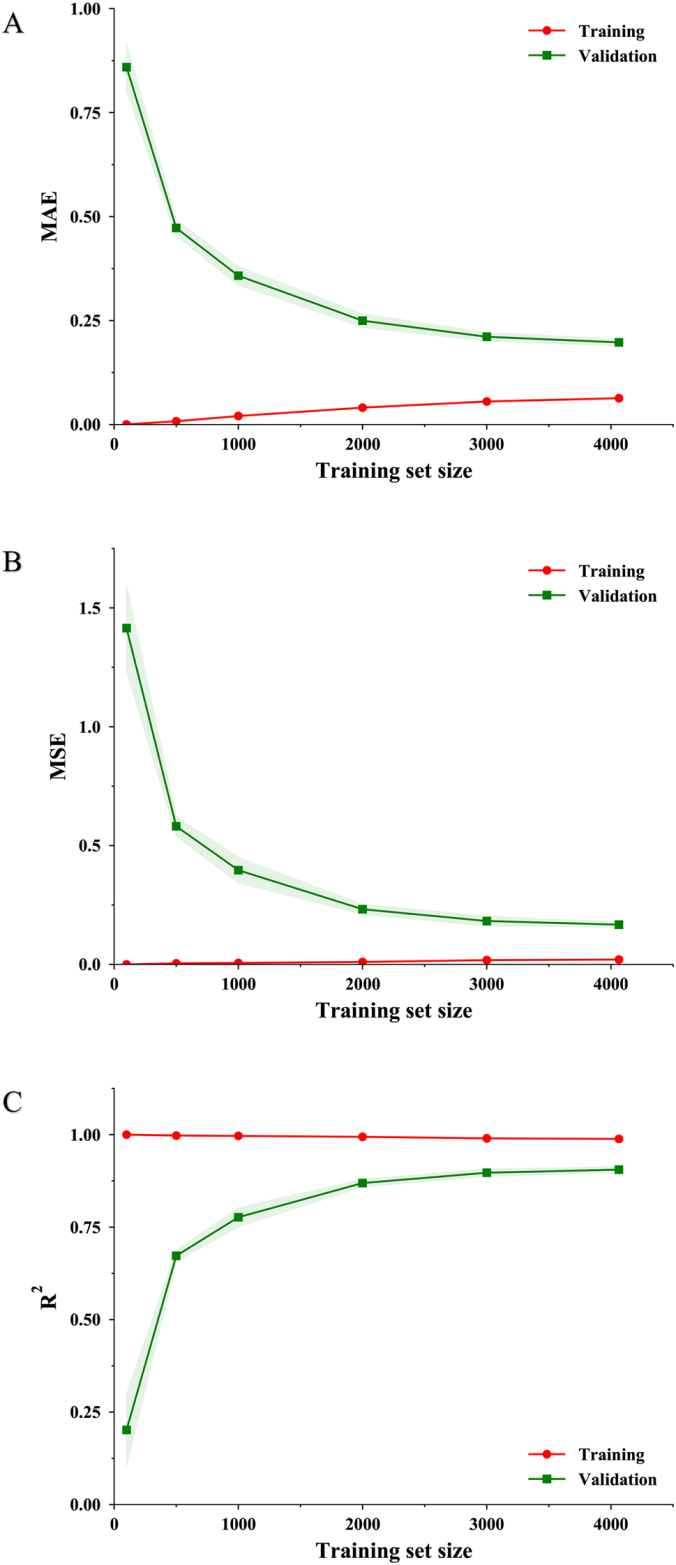
Learning curves measured by MAE (**A**), MSE (**B**), R^2^ (**C**) of lightGBM models trained on the solubility datasets with the training sizes of 100, 500, 1000, 2000, 3000 and 4064

The performance of learning algorithms is correlated with the amount of training data. Since the lightGBM algorithm had the highest performance in the final test, the learning curves of this learning algorithm were studied. The line charts in Fig. 3 showed the learning curves of lightGBM models trained on the solubility datasets with the training sizes of 100, 500, 1000, 2000, 3000, and 4064. The five-fold cross-validation method was carried out. 4/5 data were served as training data in the dataset, rest of 1/5 data as validation data at each iteration. Therefore, the largest data size of training data was 4064, 4/5 data of the complete dataset. For the training sizes less than 4064, random sampling was implemented to draw training size samples from the 4064 data. In lightGBM, the regularization term of the minimum number of samples in a child leaf was reset to 2 because the learning capability of lightGBM will be restricted if this term is still set to 18 on small training data.

As the data volume varied, the model performance and the accuracy differences between the training subset and the validation subset were shown in Fig. 3. Based on the result of the learning curves, the three metrics showed a consistent trend. The model performance on the validation subset gradually improved as the data volume increased and eventually plateaued. The model has converged, the accuracy differences between the training subset and the validation subset gradually decreased and eventually remained small.

**Fig. 4:**
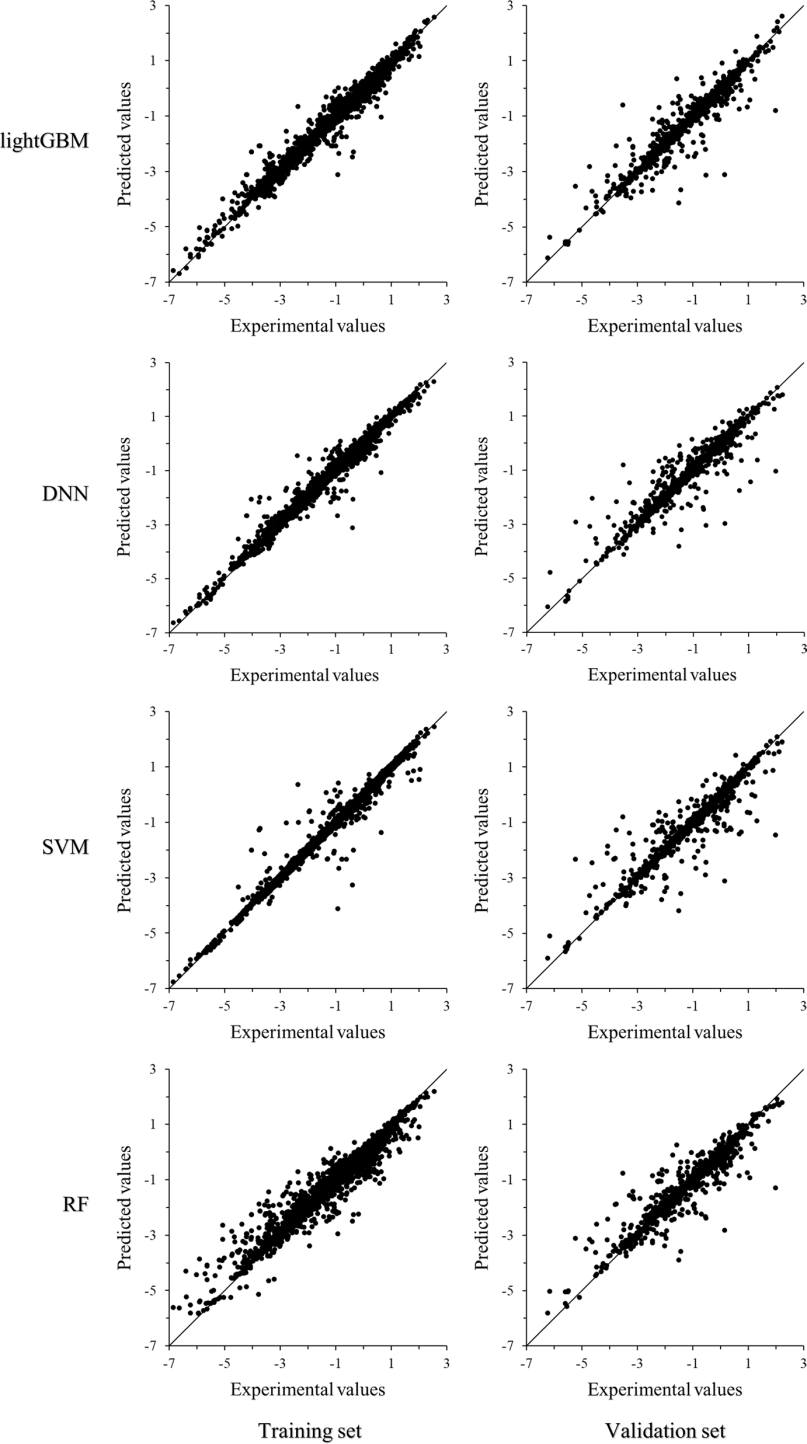
Scatter plots of lightGBM, DNN, SVM, and RF predicted values vs. experimental values of the solubility of compounds in organic solvents in the training and the validation subsets

Since the lightGBM, DNN, SVM, and RF algorithms showed relatively superior performance in the solubility dataset (Table 3), the predicted values calculated by these learning algorithms were carefully analyzed and compared. The scatter plots in Fig. 4 exhibited the predicted values calculated by lightGBM, DNN, SVM, and RF *vs.* experimental values of the solubility data in the training and the validation subsets. As shown in Fig. 4, the distribution of data points was not homogeneous. Due to the relatively intensive solubility data, the predicted values were in close proximity to the experimental values in the solubility range of − 3 to 3 for all four machine learning models. For the solubility range of − 7 to − 3, the predicted errors of DNN, SVM, and RF went up, while predictions of lightGBM reflected better generalization capabilities. Obviously, the uneven distribution of data in the dataset significantly impacted the performance of learning models. Therefore, the model robustness of lightGBM was exhibited over the solubility range of − 7 to 3 on the dataset.

The QSPR method is very general. The QSPR is primarily based on the assumption that similar molecules have similar properties [49]. It is not restricted to seen solutes or organic solvent phases in the training subset because the models investigate the underlying rules between molecule structure and solubility. The generalization ability of models in different situations was carefully analyzed. In this study, the validation subset was classified into three classes, containing (A) unseen solutes, (B) seen solutes, but not seen the same combination of solutes and solvents, and (C) seen the same combination of solute and solvent, but at different temperatures from the training subset, respectively. The corresponding data volumes for the three classes of samples in the validation subset were 20, 183, and 797, respectively.

The results in Table 4 showed that in class C, for R^2^, the accuracies of the nine machine learning models were roughly 0.8, with the lowest accuracy of 0.7779 for Ridge regression, and the highest accuracy of 0.9550 for DNN. For MSE, the accuracies of the nine machine learning models were roughly 0.2, with the lowest accuracy of 0.3762 for Ridge regression, and the highest accuracy of 0.0762 for DNN. For MAE, the accuracies of the nine machine learning models were roughly 0.25, with the lowest accuracy of 0.4408 for PLS, and the highest accuracy of 0.1216 for SVM. In class B, for R^2^, the accuracies of the nine machine learning models were roughly 0.7, with the lowest accuracy of 0.2947 for kNN, and the highest accuracy of 0.7934 for lightGBM. For MSE, the accuracies of the nine machine learning models were roughly 0.6, with the lowest accuracy of 1.4487 for kNN, and the highest accuracy of 0.4243 for lightGBM. For MAE, the accuracies of the nine machine learning models were roughly 0.55, with the lowest accuracy of 0.8734 for kNN, and the highest accuracy of 0.4729 for lightGBM. In class A, for R^2^, the accuracies of the nine machine learning models were roughly 0.2, with the lowest accuracy of − 0.7806 for kNN, and the highest accuracy of 0.4922 for lightGBM. For MSE, the accuracies of the nine machine learning models were roughly 1.3, with the lowest accuracy of 2.6338 for kNN, and the highest accuracy of 0.7511 for lightGBM. For MAE, the accuracies of the nine machine learning models were roughly 0.83, with the lowest accuracy of 1.2722 for kNN, and the highest accuracy of 0.5968 for lightGBM.

**Table 4:** Performances of machine learning algorithms in the three classes of samples in the validation subset

Machine learning algorithms	Unseen solutes			Seen solutes, but not seen the same combination of solutes and solvents			Seen the same combination of solute and solvent, but at different temperatures		
	MAE	MSE	R^2^	MAE	MSE	R^2^	MAE	MSE	R^2^
PLS	0.8467	1.2267	0.1707	0.6155	0.7112	0.6538	0.4408	0.3758	0.7781
Ridge regression	0.5909	0.8410	0.4314	0.6128	0.7123	0.6532	0.4403	0.3762	0.7779
kNN	1.2722	2.6338	-0.7806	0.8734	1.4487	0.2947	0.1919	0.1360	0.9197
DT	0.9560	1.5480	-0.0466	0.6946	0.9234	0.5505	0.2360	0.1446	0.9146
ET	0.7860	1.0640	0.2807	0.5514	0.6021	0.7069	0.1982	0.1124	0.9337
RF	0.7655	1.0518	0.2889	0.5436	0.5724	0.7213	0.1624	0.0939	0.9446
SVM	0.7959	1.3027	0.1193	0.5677	0.6375	0.6896	0.1216	0.0827	0.9512
DNN	0.8494	1.1523	0.2210	0.4897	0.5073	0.7530	0.1300	0.0762	0.9550
lightGBM	0.5968	0.7511	0.4922	0.4729	0.4243	0.7934	0.1307	0.0788	0.9535

Many solvents (60 in 123) in this data set included less than 10 solubility data. Predicting small sample data will be a challenge. In chemistry, we usually use common organic solvents to dissolve compounds. However, in some special cases, uncommon solvents are used to dissolve compounds. For example, there is no suitable common solvent, or uncommon solvents have good physicochemical properties. The accurate predictions for small data demonstrate the good performance and robustness of models in such kind of extreme cases.

The accuracies of machine learning models are tested for the uncommon organic solvents (with less than 10 data). These solubility data are in the validation subset, thus not shown in the training subset. The accuracies are shown in Table 5.

**Table 5:** Performances of machine learning algorithms for the uncommon solvents in the validation subset

Algorithm	Uncommon solvents		
	MAE	MSE	R^2^
PLS	0.5210	0.5638	0.4745
Ridge regression	0.5144	0.5574	0.4805
kNN	0.2780	0.3042	0.7165
DT	0.2792	0.2065	0.8075
ET	0.3128	0.2827	0.7365
RF	0.2568	0.2376	0.7785
SVM	0.2348	0.3139	0.7075
DNN	0.1945	0.1271	0.8815
lightGBM	0.2263	0.1796	0.8326

In Table 5, it is shown that DNN and lightGBM outperform other machine learning models for prediction of the uncommon solvents in the validation subset. In detail, for R^2^, DNN has the highest R^2^ of 0.8815, lightGBM has the second-highest R^2^ of 0.8326. For MAE and MSE, DNN has the lowest MAE and MSE of 0.1945 and 0.1271, and lightGBM has the second-lowest MAE and MSE of 0.2263 and 0.1796. This result demonstrates the good performance and robustness of DNN and lightGBM for prediction of the solubility of compounds in organic solvents. Comparing DNN with lightGBM, DNN has superior MAE, MSE and R^2^ than lightGBM for the uncommon solvents with less than 10 data. This is probably because that DNN has a deep model structure, which can be used as a general automatic feature extractor to find out the key features, thus the regressor can better approximate the underlying correlation and show a good generalizability on the small data.

### Descriptor analysis

**Fig. 5:**
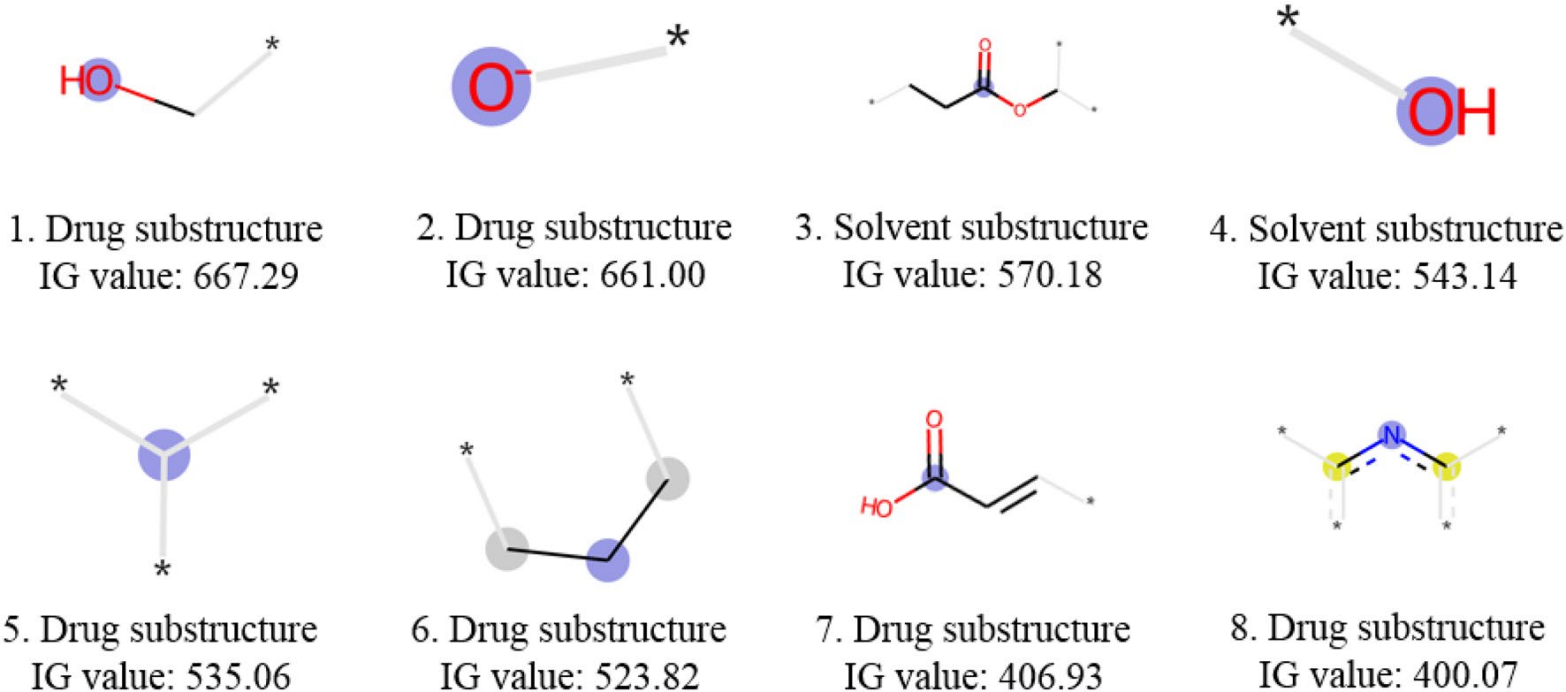
Details of the top 8 compound and solvent substructures and IG values

The model lightGBM used ECFP fingerprints to train the model, obtaining an MAE value of logS  ± 0.20. We employed the information gain (IG) values to pinpoint the important structural fragments of compounds and organic solvents on the training subset using the lightGBM program [41]. The top 8 substructures in compounds and organic solvents on the training subset data were shown in Fig. 5.

According to the results of the IG values, the important structural features contributing to the solubility prediction were identified. Structural features containing hydroxyl groups (Fig. 5, 1 and 2), carboxylic acids (Fig. 5, 7), and nitrogen-containing heterocycles (Fig. 5, 8) in the structure of compounds are likely to form hydrogen bonds with solvents. Structural features containing ester bonds (Fig. 5, 3) and hydroxyl groups (Fig. 5, 4) in the structure of solvents are likely to form hydrogen bonds with compounds. In addition, the structure of compounds may contain hydrophobic structural features, i.e., tertiary carbons (Fig. 5, 5) and chain alkanes (Fig. 5, 6).

## Discussion

A few attempts in QSPR models have been made to predict the compound solubility in organic solvents in the last 20 years. They outperformed empirical equations and mechanistic models, such as GSE and COSMO. Machine learning models were learned on the experimental solubility data using a series of machine learning algorithms and architectures. The experimental physicochemical constants, the computed topological and charge descriptors, and molecular fingerprints of compounds were used to represent the structure of molecules. For example, in 2004, Balakin et al. developed a shallow neural network to classify compounds as soluble or poorly soluble in DMSO [50]. The model was built using computed molecular descriptors containing topology, charge, and lipophilicity. 93% classification accuracy showed that the QSPR approach could predict the compound solubility in an organic solvent. In 2010, Abraham et al. proposed LFERs, often abbreviated as the Abraham model [7]. LFERs used five descriptors to represent an organic solvent and a compound, respectively. The Abraham descriptors used in the model were determined from solubility experiments. In 2019, Yousefi et al.compared shallow neural networks and SVM to predict solubility in water and organic solvents [30]. Sixty five structural group descriptors were used to generate the models. In 2020, Boobier et al. proposed to develop models by using MLR, PLS, shallow neural networks, SVM, GP, RF, and ET for solubility prediction in water, ethanol, acetone, and benzene [31]. The models were constructed on 695 ethanol data, 464 benzene data, and 452 acetone data. The results suggested that RF, ET, and SVM had the highest performance for the three organic solvents, respectively. The melting point and 14 computed descriptors used in this work were selected by human experts. However, the data used in machine learning tasks are often high-dimensional and sparse, and traditional machine learning algorithms highly depend on feature engineering. Due to the insufficient knowledge and vague experience of human experts, feature engineering is challenging and time-consuming, which ultimately leads to poor model performance. In our study, lightGBM can reduce the redundant features of raw data by bundling mutually exclusive features and improve model robustness. Deep learning can extract key features from raw data through a hierarchical architecture.

lightGBM is an ensemble procedure approach based on the GBDT framework, which has the advantages of good performance, less overfitting, high efficiency, and can handle big data. The GBDT framework is widely used in industry, and the winning solutions in many data competitions are developed based on GBDT. lightGBM is further optimized. lightGBM uses exclusive feature bundling (EFB) algorithm to bind multiple mutually exclusive features. The EFB algorithm reduces the number of features for high-dimensional sparse data and increases the computational speed of the framework. The ECFP fingerprint used in this work is composed of many 0 and 1 features. For mutually exclusive features (e.g., two features are never both 0 simultaneously), lightGBM bundles and fuses mutually exclusive features. In addition, lightGBM uses a histogram-based learning algorithm to find the best partition points for feature values. The results on different datasets show that the histogram algorithm dramatically improves both the search efficiency and the memory consumption without hurting performance, as large interval partition points suppress data noise and improve model performance [41]. Based on the histogram algorithm, lightGBM uses a leaf-wise algorithm with depth restrictions. Leaf-wise gives better accuracy than the traditional level-wise growth strategy with the same number of sample partitions. The maximum depth limit ensures time-efficient and prevents model overfitting. For these reasons, we thought lightGBM to be competitive in machine learning models.

In this study, we found that nonlinear machine learning models performed better than linear Ridge regression and PLS regression models. The relationship between solubility and the molecular structure of compounds and solvents is complicated and nonlinear. Besides lightGBM and DNN models, nonlinear SVM, RF, and ET models outperformed DT and kNN algorithms. SVM identified the support vectors and found the nonlinear hyperplane using kernel functions. RF and ET generated multiple decision trees in parallel. RF and ET integrated the predictions of multiple trees to obtain results. RF and ET could fit the nonlinear data for organic solvent solubility by constructing subtrees.

In addition to the performance of machine learning models on the overall solubility data, the model accuracies in different situations were also discussed in detail, reflecting the impact of the complexity of learning tasks on model performance. Based on the results of accuracy values (Table 4), solubility prediction at new temperature (class C) is precise and easier than the prediction for new compounds (class B) and new solvents (class A). This shows a good model generalization to temperature. For most solids, the effect of temperature on their solubility is that the solubility increases with increasing temperature [51]. This process approximates a linear relationship [52]. In addition, most of the temperature at which the solubility was determined was intensively located in the range of normal 0–60 °C (Fig. 2B), since chemical scientists are likely to choose room temperature as the experimental condition if feasible.

Performance on class B and A shows the model generalization ability to solvents and compounds, respectively. The difficulty in generalization may be due to the complexity in the highly nonlinear relationship between the solubility and substructures of compounds and solvents. For example, the substructures determine some properties like hydrogen bonds, hydrophobicity, and the free energy of solvation, which further influence the solubility of systems. In addition, the uneven data distribution of compounds and organic solvents may also contribute to the challenge in generalization. In experiments, a few compounds and organic solvents were frequently employed as the model systems (Fig. 2C, D). A few solute and solvent system solubility was repeatedly determined at different experimental temperatures in different labs. However, despite these difficulties, the present models identified key substructures (Fig. 5) from data on the one hand. Multiple key structural features synergistically contributed to the solubility. The model revealed this nonlinear relationship between solubility and structural features. On the other hand, lightGBM gave a prediction accuracy on unseen solutes (logS  ± 0.59), close to the expected noise level (logS  ± 0.4–0.7) of the experimental solubility data [6, 53, 54]. This result indicating the good performance of the present model on the unevenly distributed solubility data.

For new drug discovery and chemical synthesis, it is of significance to foresee the solubility of new compounds in organic solvents. For solvent selection, predicting the compound solubility in different solvents is important. For chemical purification, the solubility prediction of different experimental temperatures is of interest.

As mentioned above, we showed the machine learning approach for the organic solvent solubility prediction, provided a reference for the selection of organic solvents, which may accelerate solvent screening. Solubility is the maximum amount of a substance that can be dissolved by a solvent at a given physical condition [55]. Solubility is a property of chemical entities, which ranges from infinitely soluble to poorly soluble. Solubility depends on the physical and chemical properties of the solute and solvent. A basic principle is that the polarity of a solvent determines the substances it can dissolve. Substances with high polarities, such as inorganic salts, are difficult to dissolve in non-polar solvents, such as n-octanol. The polarity can be known by measuring the electric dipole moment of the substance. The emergence of various solubility prediction methods has enabled accurate solubility prediction, setting benchmarks for solvent selection in synthesis, catalysis, solubilization techniques, and insoluble active molecule development. Studies will be enabled at any stage of the chemical and pharmaceutical industry, from initial solvent screening and quality analysis to those related to formulation design. Parameters such as molecular substructure, suitable solvents, and the critical temperature for precipitation can be conceived before laboratory synthesis. Considering the importance of solubility prediction, this approach can be applied more often to predict the solubility parameters of other solvents (e.g., solvent mixtures and ionic liquids).

In addition to solubility prediction, future attempts at more in silico modeling and machine learning may go beyond solubility prediction. Thus, in silico modeling and machine learning can be used to solve various problems such as solvent selection, medical diagnostics, and protein structure prediction. Computational modeling is likely to become an essential part of the chemical and drug development process in the near future.

## Conclusions

In conclusion, this paper successfully developed a method to predict the solubility of compounds in organic solvents using the lightGBM algorithm. Ultimately, the lightGBM model exhibited, compared to deep learning and other traditional machine learning algorithms, significantly better generalization. For unseen solutes, our model gave the prediction accuracy (logS  ± 0.59) close to the expected noise level of experimental solubility data, because lightGBM can bundle mutually exclusive features to select features and utilize large separation points to generate robust models. Our method revealed the physicochemical relationship between solubility and structural features, achieving rapid solvent screening in chemistry and drug development, and may be applied to solubility prediction in other solvents in the future.

## Data Availability

The web server is free to use at https://pharmes.computpharm.org/calclogs/solubility-index.
